# The Combined Extract of Black Sticky Rice and Dill Improves Poststroke Cognitive Impairment in Metabolic Syndrome Condition

**DOI:** 10.1155/2019/9089035

**Published:** 2019-02-26

**Authors:** Warin Ohnon, Jintanaporn Wattanathorn, Wipawee Thukham-mee, Supaporn Muchimapura, Panakaporn Wannanon, Terdthai Tong-un

**Affiliations:** ^1^Department of Physiology and Graduate School (Neuroscience Program), Faculty of Medicine, Khon Kaen University, Khon Kaen 40002, Thailand; ^2^Integrative Complementary Alternative Medicine Research and Development Center, Khon Kaen University, Khon Kaen 40002, Thailand; ^3^Human High Performance and Health Promotion Research Institute, Khon Kaen University, Khon Kaen 40002, Thailand; ^4^Department of Physiology, Faculty of Medicine, Khon Kaen University, Khon Kaen 40002, Thailand

## Abstract

Despite the increase in cognitive deficit following stroke in metabolic syndrome (MetS) condition, the therapeutic strategy is still limited. Since oxidative stress and neuroinflammation play the crucial roles on the pathophysiology of aforementioned conditions, the cognitive enhancing effect of the combined extract of *Oryza sativa* and *Anethum graveolens* was considered based on their antioxidant, anti-inflammation, and neuroprotective effects together with the synergistic effect concept. Male Wistar rats weighing 180-220 g were induced metabolic syndrome-like condition by using a high-carbohydrate high-fat diet (HCHF diet). Then, reperfusion injury following cerebral ischemia was induced by the occlusion of right middle cerebral artery and treated with the combined extract of *O. sativa* and *A. graveolens* (OA extract) at doses of 0.5, 5, and 50 mg/kg BW once daily for 21 days. Spatial memory was assessed every 7 days throughout the experimental period. At the end of the study, neuron and glial fibrillary acidic protein- (GFAP-) positive cell densities, the oxidative stress status, AChE, and the expression of proinflammatory cytokines (TNF-*α*, IL-6) in the hippocampus were determined. The results showed that OA extract at all doses used in this study significantly improved memory together with the reductions of MDA, TNF-*α*, IL-6, AChE, and density of GFAP-positive cell but increased neuron density in the hippocampus. Taken together, OA is the potential cognitive enhancer in memory impairment following stroke in MetS condition. The possible underlying mechanism may occur partly via the reductions of oxidative stress status, GFAP-positive cell density, and neuroinflammatory cytokines such as TNF-*α* and IL-6 together with the suppression of AChE activity in the hippocampus. This study suggests that OA is the potential functional ingredient to improve the cognitive enhancer. However, further clinical research is required.

## 1. Introduction

Currently, the prevalence of metabolic syndrome (MetS), a cluster of metabolic disorders, is continually rising to 39-46% in every ethnic and age group [1]. It has been reported that this prevalence is increasing with the advanced age [2]. Accumulative lines of evidence during the last decade reveal that MetS is closely associated with stroke risk [3–5]. It has been demonstrated that the adjusted risk ratios for incident ischemic stroke associated with MetS are in the range of 2.1-2.47 [6–9]. Stroke is regarded as the important cause of disability. A high proportion of stroke survivors develop cognitive impairment within 3 months after stroke [10]. This defect produces the great impact on the quality of life of the patients. Accumulative lines of evidence demonstrate that oxidative stress imbalance and neuroinflammation play the crucial role on the pathophysiology of MetS, stroke, and cognitive impairment [11–16]. Based on the important role of both oxidative stress and inflammation mentioned earlier, they were considered as the target for neuroprotection.

According to the traditional folklore, food is served not only as a source of nutrients and energy but also as the tool for promoting health. Recently, food polyphenol intervention has been considered as the potential neuroprotection food. The recent study reveals that a polyphenol especially anthocyanin-rich diet can improve brain damage in an animal model of MetS induced by a high-carbohydrate high-fat diet via the attenuation of brain oxidative stress status [17] and inflammation [18]. Both *Oryza sativa*, *L. indica* (black sticky rice), and *Anethum graveolens* Linn. (dill) are commonly consumed in Thailand. They exhibit antioxidant [19, 20] and anti-inflammation effects [21, 22]. In addition, they also possess neuroprotective effect [23–25]. Based on the pharmacological effects of both herbs mentioned earlier and a synergistic effect according to the traditional folklore, we hypothesized that the combined extract of black sticky rice and dill should provide neuroprotection against stroke in an animal model of MetS. Due to the lack of available data, we aimed to determine the neuroprotective of the combined extract of black sticky rice and dill against cerebral ischemia in an animal model of MetS induced by a high-carbohydrate high-fat diet (HCHF diet). The possible underlying mechanisms were also explored.

## 2. Materials and Methods

### 2.1. Plant Material Preparation and Extraction

*Oryza sativa*, *L. indica*, and *Anethum graveolens* Linn. were collected from Khon Kaen Province. They were harvested during September-October. They were authenticated by Associate Panee Sirisa-ard, pharmacognosy expert from Faculty of Pharmacy, Chiang Mai University, who served as the consultant of High Human Performance and Health Promotion Research Institute, Khon Kaen University, Thailand. Voucher specimens (ICAM 12001 and ICAM12002) were deposited at Integrative Complementary Alternative Medicine Research and Development Center, Khon Kaen University. For the preparation, the grains of black sticky rice and aerial part of dill were cleaned and dried in the oven (Memmert GmbH, USA) at 60°C for 72 hours. Then, the water extract of black sticky rice and 95% hydroalcoholic extract were prepared by using maceration technique. In brief, the plant materials mentioned earlier were subjected to the 24-hour maceration at room temperature. The extracts were harvested, centrifuged at 3000 rpm for 10 min, and filtered with Whatman no. 1 filter paper. The filtrates were collected and subjected to a freeze drying process. The percentage yields of *O. sativa* and *A. graveolens* were 10 and 26, respectively. The yielded extracts were stored at -20°C until use. The combination extract (OA extract) was prepared by mixing *O. sativa* and *A. graveolens* at a ratio of 1 : 6 based on our pilot data which showed that this ratio showed the highest potential for treating cerebral ischemia in metabolic syndrome.

### 2.2. Measurement of Total Phenolic Compound Contents

The total phenolic content of OA extract was determined by using the Folin-Ciocalteu colorimetric method. In brief, 1000 *μ*l of a 50% Folin-Ciocalteu phenol reagent (Sigma-Aldrich, USA) and 158 *μ*l of distilled water were mixed with 20 *μ*l of the tested substances (OA extract and gallic acid) and incubated at 37°C for 8 minutes. At the end of the incubation period, 30 *μ*l of 20% Na_2_CO_3_ (Sigma-Aldrich, USA) was added, mixed, and incubated at room temperature in the dark room for 2 hours. Then, the absorbance at 765 nm was recorded. Results were expressed as mg gallic acid equivalent (GAE)/mg per 0.1 g of OA extract. Various concentrations of gallic acid (Sigma-Aldrich, USA) ranging from 0 to 500 *μ*g/ml were used to prepare the standard calibration curve [26].

### 2.3. Measurement of Flavonoid Content

The modified aluminium chloride colorimetric method was used to measure the flavonoid content of OA extract [27]. Aliquots of 1.5 ml of the tested substances were added to equal volumes of a solution of 2% AlCl_3_·6H_2_O (2 g in 100 ml methanol). Then, the mixture was subjected to a vigorous shake and incubated at room temperature for 30 minutes. At the end of the incubation period, an absorbance at 415 nm was measured. The contents of flavonoids were expressed as *μ*g quercetin equivalent/mg extract.

### 2.4. Antioxidant Property Assessment

#### 2.4.1. 1,1-Diphenyl-2-picryl-hydrazyl (DPPH) Radical Assay

This method measured an antioxidant activity of the tested substance on the basis of the scavenging activities of the stable 1,1-diphenyl-2-picrylhydrazyl (DPPH) radical [28]. Briefly, 0.25 ml of 0.15 mM DPPH solution was mixed with 50 *μ*l of OA extract solution of varying concentrations (1, 5, 10, 50, 100, 250, 500, and 1000 *μ*g/ml). After a 30-minute incubation period in a dark condition, an absorbance at 517 nm was recorded using a Spectronic™ GENESYS™ 20 spectrophotometer (Thermo Electron Corporation, IL, USA). Corresponding blank was prepared, and L-ascorbic acid was used as standard reference. Extract concentration which provided 50% inhibition (IC50) was calculated using the graph by plotting inhibition percentage against extract concentration [28, 29].

#### 2.4.2. Ferric Reducing Antioxidant Power (FRAP) Assay

The antioxidant potential to change ferric tripyridyltriazine (Fe^3+^-TPTZ) complex to ferrous tripyridyltriazine (Fe^2+^-TPTZ) of OA extract was measured by using FRAP assay. FRAP reagent was freshly prepared by mixing 25 ml of 300 mM acetate buffer (Sigma-Aldrich, USA), pH 3.6, and 2.5 ml of 10 mM TPTZ (Sigma-Aldrich, USA) solution in 2.5 ml of 20 mM ferric chloride solution (FeCl_3_) (Sigma-Aldrich, USA) together. Then, 190 *μ*l of the FRAP reagent and 100 *μ*l of distilled water were mixed with 10 *μ*l of OA extract at various concentrations ranging from 1 to 1000 *μ*g/ml. The mixture was incubated at 37°C for 10 minutes. After the incubation, an absorbance was recorded at 593 nm [30]. Ascorbic acid was used as the positive control, and results were expressed as the EC50 value.

### 2.5. Measurement of Acetylcholinesterase Inhibitory (AChEI) Activity

AChE suppression activity of OA extract was determined by a colorimetric method according to the method of Ellman et al. [31]. This method is based on the determination of a yellow color of 5,5′-dithiobis (2-nitrobenzoic acid) produced by the hydrolysis of acetylcholine by acetylcholinesterase (AChE). In brief, various concentrations of OA extract at the volume of 25 *μ*l each were added to the reaction mixture containing 50 *μ*l of Tris-HCl (50 mM, pH 8.0) (Sigma-Aldrich, USA), 75 *μ*l of 3 mM 5,5′-dithio-bis-2-nitrobenzoic acid (DTNB) (Sigma-Aldrich, USA), 25 *μ*l of 15 mM thiocholine iodide (ATCI) (Sigma-Aldrich, USA), and 25 *μ*l of AChE (0.22 U/ml) (Sigma-Aldrich, USA). After mixing, the reaction mixture was incubated at room temperature for 5 minutes, the absorbance at 412 nm was recorded with a microplate reader (iMark™ Microplate Absorbance Reader). Percentage of inhibition was calculated by comparing the rate of enzymatic hydrolysis of ATCI for the samples to that of the blank (50% aqueous methanol in buffer). Donepezil (1-32 mM) (ARICEPT®, USA) was used as a reference standard. The AChE inhibition activity of each sample was expressed in terms of EC50. Every sample was assessed in triplicate.

### 2.6. Determination of Cyclooxygenase-2 (COX-2) Activity

The activity of cyclooxygenase-2 (COX-2), an enzyme playing an important role in inflammatory event, was assessed. Briefly, the reaction mixture containing 150 *μ*l of 100 mM Tris-HCl buffer, 10 *μ*l 0.5 *μ*M of heme, 10 *μ*l of 50 nM COX-2, and 10 *μ*l of OA extract was mixed in 96-well microliter plates. The reaction was initiated with 20 *μ*l of 100 *μ*M arachidonic acid and 20 *μ*l of 10 *μ*M of TMPD (N,N,N′,N′-tetramethyl-p-phenylenediamine) (Cayman Chemical, USA). Then, the plate was incubated at room temperature for 5 minutes. The absorbance at 590 nm was measured at the end of the incubation period by using a microplate reader. Indomethacin was used as a standard compound. The percentage of COX-2 inhibition was calculated, and results were expressed as the EC50 value [32].

### 2.7. Finger Print Chromatogram Assessment

The phenolic profiles of OA extract consisting of cyanidin-3-glucoside (Sigma-Aldrich, USA), gallic acid (Sigma-Aldrich, USA), and quercetin-3-O-rutinoside (Sigma-Aldrich, USA) were determined by high-performance liquid chromatography (HPLC). Chromatography was performed by using a Waters® system equipped with a Waters® 2998 photodiode array detector. Chromatographic separation was performed using Purospher® STAR, C-18 encapped (5 *μ*m), LiChroCART® 250-4.6, and HPLC-Cartridge, Sorbet Lot no. HX255346 (Merck, Germany). The mobile phase (HPLC grade) consisted of 100% methanol (solvent A) (Fisher Scientific, USA) and 2.5% acetic acid (solvent B) (Fisher Scientific, USA) in deionized (DI) water was used to induce gradient elution. The gradient elution was carried out at a flow rate of 1.0 ml/min with the following gradient: 0-17 min, 70% A; 18-20 min, 100% A; and 20.5-25 min, 10% A. The sample was filtered (0.45 *μ*m, Millipore), and a direct injection of the tested sample at the volume of 20 *μ*l on the column was performed. The chromatograms were recorded at 280 nm using the UV detector, and data analysis was performed using Empower™ 3.

### 2.8. Experimental Protocol

Adult male Wistar rats weighing 180-220 grams at the ages between 10 and 14 weeks were obtained from National Laboratory Animal Center, Salaya, Nakhon Pathom, Thailand. The animals were housed in a group of 6 per cage in the standard metal cages at 22 ± 2°C on a 12 : 12 h light : dark cycle and ad libitum access to food and water. All experimental protocols used in this study had been approved by the Institutional Animal Care and Use Committee, Khon Kaen University, Khon Kaen, Thailand (AEKKU 30/2558). After 1 week of acclimatization, rats were divided into seven groups (*n* = 6) as follows:

1. Group I (naïve intact): rats in this group received a normal diet (4.5% fat, 42% carbohydrate, and 24% protein) and received no treatment
2. Group II (HCHF+sham operation+vehicle): all rats in this group received a HCHF diet and subjected to sham operation and vehicle treatment
3. Group III (HCHF+MCAO+vehicle): animals in this group were HCHF diet-treated rats which were subjected to reperfusion after the occlusion of right middle cerebral artery (Rt. MCAO) and treated with vehicle
4. Group IV (HCHF+MCAO+vitamin C): all animals were HCHF diet-treated rats which were subjected to reperfusion after the occlusion of right middle cerebral artery (Rt. MCAO) and treated with vitamin C at dose of 250 mg/kg BW
5. Groups V-VII (HCHF+MCAO+OA) (OA1, OA2, and OA3): all rats in these groups were HCHF diet-treated rats which were exposed to reperfusion injury after the occlusion of right middle cerebral artery (Rt. MCAO) and treated with OA extract at various doses ranging from 0.5, 5, to 50 mg/kg BW

**Figure 1:**
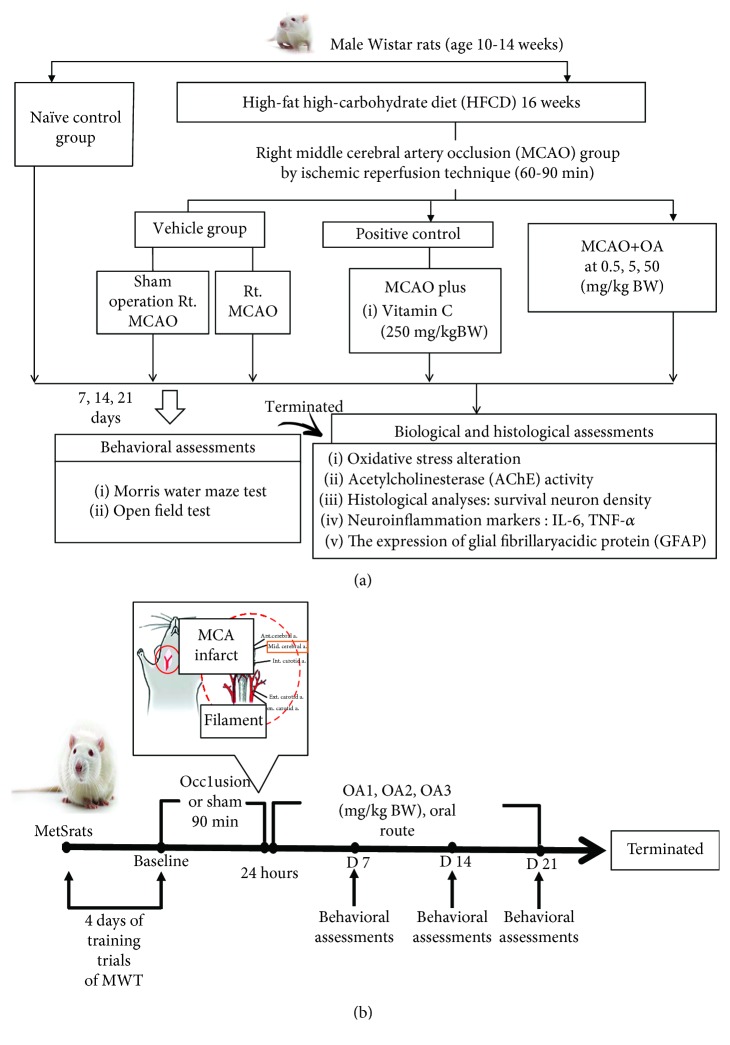
Schematic diagram showing all experimental procedures. (a) Experimental protocol of OA extract treatment and the determination of various parameters. (b) Right MCAO induction and schedule for OA extract treatment. IL-6: interleukin-6; TNF-*α*: tumor necrosis factor-*α*; HCHF: high- high-fat diet; MCAO: right middle cerebral artery occlusion; OA1, OA2, and OA3: the combined extract of *O. sativa* and *A. graveolens* at doses of 0.5, 5, and 50 mg/kg BW, respectively.

Rats in group II-group VII were fed a high-carbohydrate high-fat diet (HCHF; 35.83% fat, 35.54% carbohydrate, and 28.63% protein) in order to induce metabolic syndrome. After 16 weeks of the feeding period, rats which showed the percentage change of body weight more than 40 percent, fasting plasma glucose more than 100 mg/dl, systolic blood pressure more than 130 or diastolic blood pressure more than 85 mmHg, and the atherosclerosis index (total serum cholesterol/total serum HDL-C) higher than the control group were selected for inducing reperfusion injury after the occlusion of Rt. MCAO. Then, the animals were orally given the assigned substances once daily for 21 days. Spatial memory was assessed by using the Morris water maze test every 7 days throughout a 21-day study period. At the end of the study period, neuron density, the oxidative stress status, AChE, density of glial fibrillary acidic protein- (GFAP-) positive cell, and the expressions of proinflammatory cytokines (TNF-*α*, IL-6) in the hippocampus were determined. The schematic diagram showing the experimental protocol was shown in Figure 1.

### 2.9. Focal Cerebral Ischemia/Reperfusion Induction

The rats were anesthetized with an intraperitoneal injection of pentobarbital sodium (50 mg/kg BW; Tianjin Kemiou Chemical Reagent Co. Ltd., Tianjin, China). Then, the monofilament was inserted via the right common carotid artery, and the right external carotid artery (ECA) was exposed through a ventral midline neck incision and was ligated proximally. A silicone-coated nylon monofilament (4-0) suture (USS DG; Tyco Healthcare Group LP, CT, USA), with its tip rounded by heating near a flame, was inserted 17-18 mm into the right internal carotid artery (ICA) from distal to the carotid bifurcation, to occlude the origin of the MCA. The suture was fixed, and the incision was closed. Following 90 min of ischemia, the nylon suture was withdrawn to allow reperfusion. The sham-operated rats underwent identical surgery, but the nylon suture was not inserted. After 1 week of operation, rats in groups V-VII were subjected to the assigned treatments once daily for a period of 21 days.

### 2.10. Spatial Memory Assessment

Spatial memory was assessed by using the Morris water maze test. The water maze apparatus is a circular pool at the diameter of 147 cm and filled with tap water to the depth of 40 cm. The surface was covered with nontoxic powder. The pool was divided into 4 equal quadrants, and the removable escape platform was placed in the center on one quadrant below the water level. The location of the platform was invisible, and it remained there throughout the training. Each animal was trained to memorize the location of the platform by forming the association information between its location and the location of platform by using external cues. After 4 training sessions, animal was placed in the water in the starting quadrant and allowed to swim until the animal found and climbed onto the platform. The time which the animal spent to reach the hidden platform was recorded as escape latency. The retention time was determined 24 h later by exposing the animal to subject to the same situation as mentioned earlier except that the immersed platform was removed and the time which the animal spent for swimming in the quadrant previously located platform was regarded as retention time [25].

### 2.11. Locomotor Assessment

The effect of the developed product on locomotor activity was assessed by using an open field test, the most frequently used method. A 90 cm square plexiglass chamber with a 70 cm height was used for behavioral evaluation. The tests were performed in a room lit by a 60 W light bulb 1.75 m above the center of the open field. Each animal was placed into the center of the open field chamber and allowed to explore the apparatus for 5 minutes. Every time both hind paws entered one of these squares, a crossing was recorded. In addition to the number of crossing, the number of center square entries was also recorded. The number of licking, rearing, and grooming was also monitored by using a video tracking system [33].

### 2.12. Histological Study

#### 2.12.1. Nissl Staining

For histopathological analysis, the brains were perfused transcardially with fixative solution containing 4% paraformaldehyde (Sigma-Aldrich, USA) in 0.1 M phosphate buffer pH 7.4 overnight at 4°C. Then, they were infiltrated with 30% sucrose (Merck, Germany) solution for 72 h at 4°C. Serial sections of tissues were cut frozen on a cryostat (Thermo Scientific™ HM 525 Cryostat) at 20 *μ*m thick. All sections were picked up on slides coated with 0.3% aqueous solution of gelatin containing 0.05% aluminium potassium sulfate (Sigma-Aldrich, USA). The triplicate coronal sections of brains were immersed in 0.2% cresyl violet (Sigma-Aldrich, USA) for 8 minutes, rinsed with double distilled water, and dehydrated through graded alcohols (70, 95, and 100% 2x) (RCI Labscan, Thailand). The sections were cleared with xylene for 5 minutes (2 times) and mounted using DPX mountant (Merck, Germany). The evaluation of neuron density in the hippocampus was performed under an Olympus light microscope model BH-2 (Japan) at 40x magnification. Counts were performed in three adjacent fields, and the mean number was calculated and expressed as density of neurons per 255 *μ*m^2^.

#### 2.12.2. Immunohistochemistry

Brain sections containing the hippocampus were prepared as mentioned in Section 2.12.1. All sections were picked up on slides coated with 0.3% aqueous solution of gelatin containing 0.05% aluminium potassium sulfate (Sigma-Aldrich, USA). The sections were heated using a microwave oven in 0.01 M sodium citrate buffer (pH 6.0) for 10 minutes and cooled at room temperature. The sections were subjected to a 5-minute washing step with phosphate-buffered saline (PBS) for 3 times and incubated in 0.3% hydrogen peroxide at room temperature for 20 minutes. At the end of the incubation period, the sections were subjected to a 5-minute washing with PBS for 3 times. Then, they were incubated in the mixture containing 0.3% Triton X-100 (Fluka Chemika, Buchs, Switzerland), 1% (*w*/*v*) bovine serum albumin (BSA), and 10% normal goat serum at room temperature for 20 minutes. Following this process, the sections were washed with PBS for three times (5 minutes each) and incubated with primary anti-GFAP (Abcam, Cambridge, MA, USA) at a dilution of 1 : 500 (diluted in the solution containing 0.01 M PBS with 1% Triton X-100 and 10% normal serum) at 4°C overnight. Following this step, the sections were washed and incubated with REAL™ EnVision™ Detection System, Peroxidase/DAB+ rabbit/mouse (Dako, Glostrup, Denmark) at room temperature for 30 minutes. At the end of the incubation period, the sections were rinsed with PBS and incubated with 3,3′-diaminobenzidine tetrahydrochloride (DAB) (Sigma-Aldrich, USA) for 5 minutes. Positive staining was recognized as a brown color. Negative control sections were subjected to the same procedures except the exposure to primary antibody. The sections were mounted on gelatin-coated slides, counterstained with cresyl violet, dehydrated with graded alcohols, cleared with xylene, and mounted with DPX mountant. The numbers of positive cells in an area of 255 *μ*m^2^ were counted. The data were shown as mean and standard error. Cell counts were carried out by a technician who is blind to the experimental design. All measurements were repeated for three times, and the mean value was used.

### 2.13. Brain Homogenate Preparation

Hippocampi were isolated, weighed, and homogenized with a buffer consisting of 50 volume of 0.1 M phosphate-buffered saline. Then, the brain homogenates were centrifuged at 3000*g* for 15 min at 4°C. Supernatant of tissue homogenates was collected and used for the determinations of acetylcholinesterase (AChE) and oxidative stress markers including malondialdehyde (MDA) and the activities of main scavenger enzymes such as catalase (CAT), superoxide dismutase (SOD), and glutathione peroxidase (GSH-Px). The protein concentration in brain homogenate was also determined by using a Thermo Scientific NanoDrop 2000c spectrophotometer (Thermo Fisher Scientific, Wilmington, Delaware, USA).

### 2.14. Biochemical Assessments

#### 2.14.1. Oxidative Stress Marker Assessment

The level of malondialdehyde (MDA), a lipid peroxidation product, in the brain was determined by determining the accumulation of thiobarbituric acid reactive substances (TBARSs) according to the method of Ohkawa et al. and Harishekar and Kiran [34, 35]. In brief, 50 *μ*l of sample was mixed with the solution containing 50 *μ*l of 8.1% sodium dodecyl sulphate (SDS) (Sigma-Aldrich, USA), 375 *μ*l of 0.8% of thiobarbituric acid (TBA) (Sigma-Aldrich, USA), 375 *μ*l of 20% acetic acid (Sigma-Aldrich, USA), and 150 *μ*l of distilled water (DW). Then, the mixture was boiled in a water bath at 95°C for 1 hour and cooled immediately under tap water. Then, 250 *μ*l of DW and 1250 *μ*l of the solution containing n-butanol and pyridine (15 : 1 *v*/*v*) (Merck, Germany) were added and mixed together and centrifuged at 4000 rpm for 10 minutes. The upper layer was separated, and the absorbance at 532 nm was measured. 1,3,3-Tetramethoxy propane (0-15 *μ*M) (Sigma-Aldrich, USA) was used as the standard, and the level of MDA was expressed as ng/mg protein.

SOD activity was monitored based on the inhibition of nitroblue tetrazolium (NBT) reduction [36]. In brief, 20 *μ*l of sample was mixed with the reaction mixture containing 57 mM phosphate buffer solution (KH_2_PO_4_) (Sigma-Aldrich, USA), 0.1 mM EDTA (Sigma-Aldrich, USA), 10 mM cytochrome C solution (Sigma-Aldrich, USA), 50 *μ*M of xanthine solution, and 20 *μ*l of xanthine oxidase solution (0.90 mU/ml) at 25°C. The absorbance at 415 nm was measured. SOD enzyme (Sigma-Aldrich, USA) activities at the concentrations of 0-25 units/ml were used as the standard, and the results were expressed as units/mg protein.

Catalase activity measurement was carried out spectrophotometrically by measuring the decrease in absorbance of H_2_O_2_ [37]. In brief, 50 *μ*l of 30 mM hydrogen peroxide (in 50 mM phosphate buffer, pH 7.0) (BDH Chemicals Ltd., UK), 25 *μ*l of H_2_SO_4_ (Sigma-Aldrich, USA), 150 *μ*l of 5 mM KM_n_O_4_ (Sigma-Aldrich, USA), and 10 *μ*l of tissue homogenate were mixed thoroughly, and changes in an absorbance of the reaction solution at 490 nm were determined. CAT enzyme (Sigma-Aldrich, USA) at the concentration range of 0-100 units/ml was used as the standard, and the result was expressed as units/mg protein.

In order to determine the activity of GSH-Px, the mixture containing 10 *μ*l of 1 mM dithiothreitol (DTT) (Sigma-Aldrich, USA) in 6.67 mM potassium phosphate buffer (pH 7), 100 *μ*l of 1 mM sodium azide (Sigma-Aldrich, USA) in 6.67 mM potassium phosphate buffer (pH 7), 10 *μ*l of 50 mM glutathione (Sigma-Aldrich, USA) solution, 100 *μ*l of 30% hydrogen peroxide (BDH Chemicals Ltd., UK), and 20 *μ*l of sample solution was mixed and incubated at room temperature for 5 minutes. Then, 10 *μ*l of 10 mM DTNB (5,5-dithiobis-2-nitrobenzoic acid) (Sigma-Aldrich, USA) was added, and the optical density at 412 nm was recorded at 25°C over a period of 5 min [38]. The standard calibration curve was prepared by using GSH-Px enzyme (Sigma-Aldrich, USA) at the concentration range of 0-5 units/ml. GSH-Px activity was expressed as units/mg protein.

#### 2.14.2. In Vivo Assessment of AChE

The activity of rat brain AChE was determined by the method of Ellman with a slight modification [31]. In brief, a mixture containing a 20 *μ*l of sample solution, 200 *μ*l of 0.1 mM sodium phosphate buffer (pH 8.0) (Sigma-Aldrich, USA), and 10 *μ*l of 0.2 M DTNB (5,5′-dithio-bis-(2-nitrobenzoic acid)) (Sigma-Aldrich, USA) was mixed and incubated at room temperature for 5 minutes. Then, 10 *μ*l of 15 mM acetylcholine thiochloride (ACTI) (Sigma-Aldrich, USA) was added and incubated for 3 minutes. The change in absorbance of light was measured at 412 nm for 3 minutes at regular intervals of 30 seconds using a microplate reader (iMark™ Microplate Absorbance Reader). The activity of AChE was calculated according to the equation below and expressed as nmol/min·mg protein.

(1) $$\begin{array}{c} \text{AChE activity}=\left(\frac{\Delta A}{\left(1.36\times 10^{4}\right)}\right)\times \left(\frac{1}{\left(20/230\right)}\right)C, \end{array}$$

where Δ*A* = the difference of absorbance/minute and *C* = protein concentration of brain homogenate.

### 2.15. Western Blotting Analysis

The hippocampus was homogenized in the mammalian protein extraction reagent (M-PER; Pierce Protein Biology Product, Rockford, IL, USA), with protease inhibitor cocktail (1 : 10) (Sigma-Aldrich, USA). Then, the samples were centrifuged at 12,000*g* for 10 minutes at 4°C, and the supernatant was collected and stored on ice for protein determination by using a Thermo Scientific NanoDrop 2000c spectrophotometer (Thermo Fisher Scientific, Wilmington, Delaware, USA). For each animal, 80 *μ*g of hippocampal lysate was adjusted to appropriate concentration by using Tris-Glycine SDS-PAGE loading buffer (Bio-Rad, USA) and heated at 95°C for 10 minutes. In addition, 20 *μ*l of tissue sample protein was loaded onto SDS-polyacrylamide gel and separated by sodium dodecyl sulfate-polyacrylamide gel electrophoresis (SDS-PAGE). Biotinylated broad-range molecular weight markers (Bio-Rad) were loaded onto the gels as well. After electrophoretic separation, proteins were transferred to a nitrocellulose membrane by electroblotting, washed with 0.05% TBS-T, and incubated overnight at 4°C in blocking buffer (PBS containing 1% Tween-20 (T-PBS) and 6.5% nonfat dry milk). Membranes were then incubated overnight at 4°C with polyclonal rabbit TNF-*α* (#3707) and IL-6 (#12912) primary antibodies (Cell Signaling Technology, USA; dilution 1 : 1000), rinsed with T-PBS for 30 minutes, and incubated with anti-rabbit IgG, HRP-linked antibody (Cell Signaling Technology, USA; dilution 1 : 2000) at room temperature for 1 hour. The bands were visualized and quantitated by using the ECL detection systems (GE Healthcare) and LAS-4000 luminescent image analyzer (GE Healthcare). Band intensities were measured for statistical analysis using the ImageQuant TL v.7.0 image analysis software (GE Healthcare). The expression was normalized using *β*-actin (Cell Signaling Technology, USA; dilution 1 : 2000). Data were presented as a relative density to the control normal group.

### 2.16. Statistical Analysis

All data are expressed as mean ± standard error of mean (SEM). Statistical significance was evaluated by using one-way analysis of variance (ANOVA), followed by the post hoc (Tukey) test. The statistical significance was regarded at *P* values < 0.05.

## 3. Results

### 3.1. Characterization of the Combined Extract of *O. sativa* and *A. graveolens* (OA Extract)

The characterization of the combined extract of *O. sativa* and *A. graveolens* or OA extract was shown in Table 1. *O. sativa* extract and *A. graveolens* contained the phenolic compounds at the concentrations of 824.62 ± 90.98 and 941.54 ± 41.63 *μ*g GAE/mg extract while the concentration of this substance in the combined extract of *O. sativa* and *A. graveolens* (OA extract) was 1724.10 ± 159.73 *μ*g GAE/mg extract. In addition, the flavonoid contents in *O. sativa* and *A. graveolens* were 47.40 ± 2.98 and 23.81 ± 0.32 *μ*g quercetin/mg extract, respectively. It was found that the concentration of flavonoid content in OA extract was 62.18 ± 1.03 *μ*g quercetin/mg extract. The contents of phenolic compounds and flavonoids in OA extract were significantly higher than those in *O. sativa* (*P* value <0.001 all) and *A. graveolens* (*P* value <0.01 and 0.001). The antioxidant activities were assessed by using DPPH and FRAP assays. The current results showed that EC50 of *O. sativa* and *A. graveolens* via DPPH assay were 0.123 ± 0.01 and 0.065 ± 0.02 mg/ml while EC50 of both substances mentioned earlier via FRAP assay were 2.56 ± 0.37 and 2.99 ± 0.055 mg/ml, respectively. It was found that EC50 of OA extract via DPPH and FRAP assays were 0.014 ± 0.003 and 1.19 ± 0.24 mg/ml, respectively. The antioxidant activity of OA extract assessed via DPPH assay showed the significant higher potent activity than the antioxidant activity of *O. sativa* (*P* value < 0.01) while the antioxidant activity of OA extract assessed via FRAP assay showed the significant higher potent activity than the activity of both *O. sativa* and *A. graveolens* (*P* value < 0.05 all). *O. sativa* also showed that EC50 of acetylcholinesterase suppression and cyclooxygenase-2 (COX-2) suppression activities were 2.03 ± 1.08 and 9.37 ± 1.18 mg/ml, respectively. EC50 of both mentioned activities of *A. graveolens* were 2.94 ± 0.62 and 142.44 ± 15.82 mg/ml, respectively. The present data showed that EC50 of acetylcholinesterase suppression and cyclooxygenase-2 (COX-2) suppression activities of OA extract were 1.98 ± 0.15 and 1.73 ± 0.44 mg/ml, respectively. It is also clearly shown that COX-2 suppression activity of OA extract was significantly potent than that of *A. graveolens* (*P* value < 0.001).

**Figure 2:**
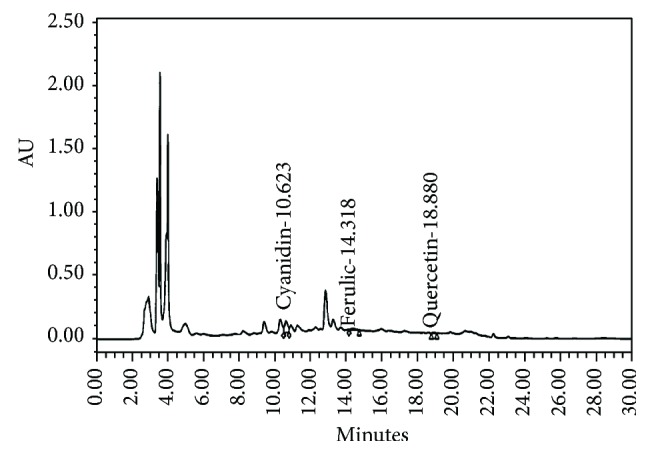
The chromatogram finger print of OA extract (100 mg/ml) using Purospher® STAR, C-18 encapped (5 *μ*m), LiChroCART® 250-4.6, and HPLC-Cartridge, Sorbet Lot no. HX255346 (Merck, Germany) with guard column (Merck, Germany).

The fingerprint chromatogram of OA extract was also determined, and data were shown in Figure 2. The contents of cyanidin-3-glucoside, quercetin, and ferulic acid in OA extract were 0.52 ± 0.003 *μ*g Cyn-3-glu/50 mg of extract, 3.00 ± 0.58 *μ*g QE/50 mg of extract, and 1.16 ± 0.12 *μ*g ferulic acid/50 mg of extract, respectively.

### 3.2. Cognitive Enhancing Effect of OA Extract

**Figure 3:**
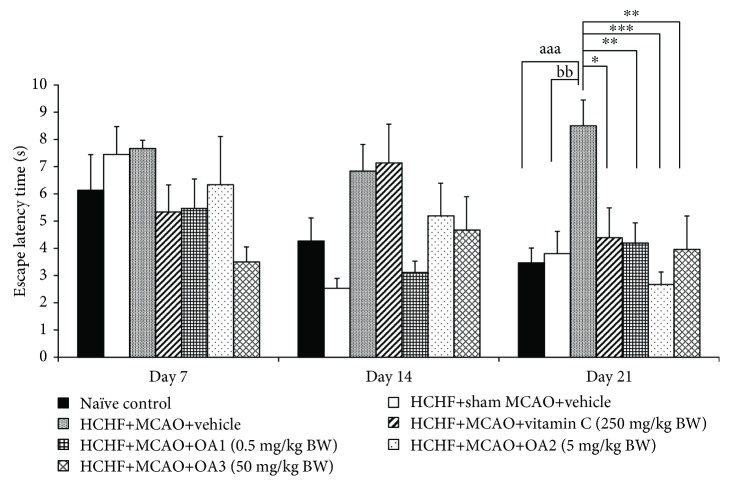
Effect of OA extract on escape latency time. Data are presented as mean ± SEM (*n* = 6/group). ^aaa^*P* value < 0.001, compared to naïve intact rats; ^bb^*P* value < 0.01, compared to sham operation which received HCHF diet and vehicle; and ^∗,∗∗,∗∗∗^*P* value < 0.05, 0.01, and 0.001, respectively, compared to MCAO rats which received HCHF and vehicle. HCHF: high-carbohydrate high-fat diet; MCAO: right middle cerebral artery occlusion; OA1, OA2, and OA3: the combined extract of *O. sativa* and *A. graveolens* at doses of 0.5, 5, and 50 mg/kg BW, respectively.

**Figure 4:**
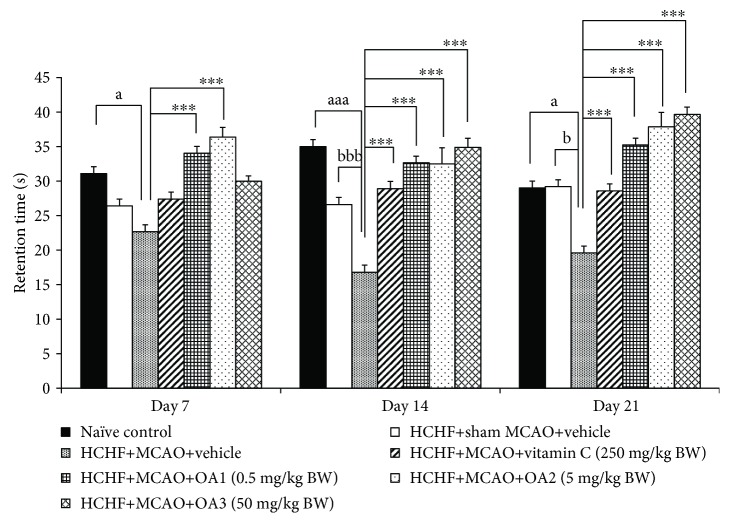
Effect of OA extract on retention time. Data are presented as mean ± SEM (*n* = 6/group). ^a,aaa^*P* value < 0.05 and 0.001, respectively, compared to naïve intact rats; ^b,bbb^*P* value < 0.01 and 0.001, respectively, compared to sham operation which received HCHF diet and vehicle; and ^∗∗∗^*P* value < 0.001, compared to MCAO rats which received HCHF and vehicle. HCHF: high-carbohydrate high-fat diet; MCAO: right middle cerebral artery occlusion; OA1, OA2, and OA3: the combined extract of *O. sativa* and *A. graveolens* at doses of 0.5, 5, and 50 mg/kg BW, respectively.

**Figure 5:**
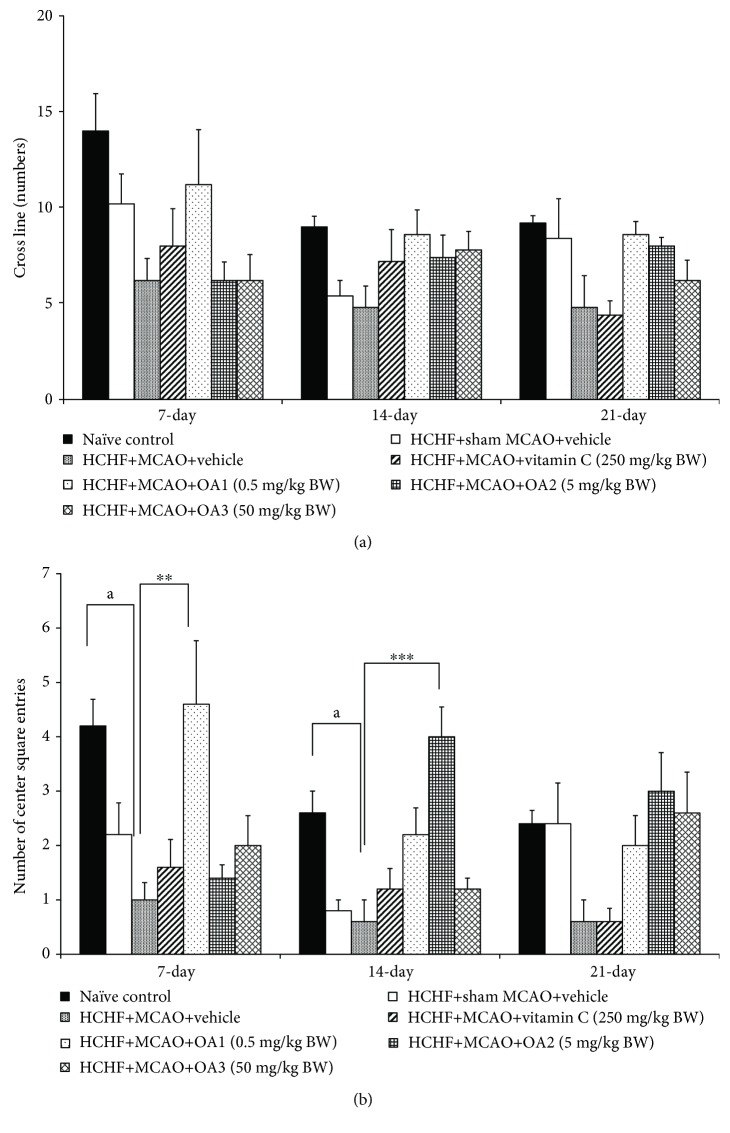
Effect of OA extract on locomotor activities: (a) the number of cross line and (b) the number of center square entries. Data are presented as mean ± SEM (*n* = 6/group). ^a^*P* value < 0.05, compared to naïve intact rats and ^∗∗,∗∗∗^*P* value < 0.01 and 0.001, respectively, compared to MCAO rats which received HCHF and vehicle. HCHF: high-carbohydrate high-fat diet; MCAO: right middle cerebral artery occlusion; OA1, OA2, and OA3: the combined extract of *O. sativa* and *A. graveolens* at doses of 0.5, 5, and 50 mg/kg BW, respectively.

**Figure 6:**
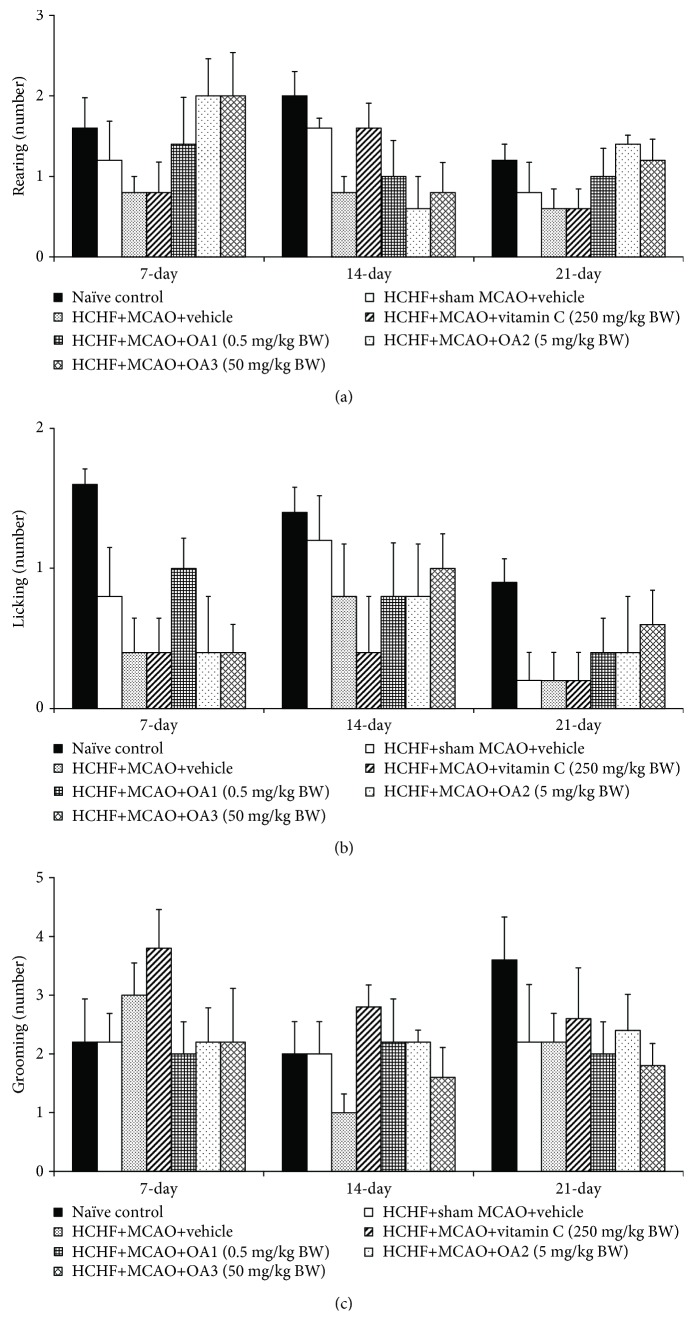
Effect of OA extract on exploratory activities: (a) licking, (b) rearing, and (c) grooming behaviors. Data are presented as mean ± SEM (*n* = 6/group). HCHF: high-carbohydrate high-fat diet; MCAO: right middle cerebral artery occlusion; OA1, OA2, and OA3: the combined extract of *O. sativa* and *A. graveolens* at doses of 0.5, 5, and 50 mg/kg BW, respectively.

Figure 3 showed that no significant changes in escape latency among various groups were observed at 7 and 14 days after MCAO. However, metabolic syndrome rats induced by a HCHF diet which subjected to MCAO and received vehicle significantly increased escape latency (*P* value < 0.01, compared to the HCHF+sham operation+vehicle group). Interestingly, this change was attenuated by vitamin C and OA extract at doses of 0.5, 5, and 50 mg/kg (*P* value < 0.05, 0.01, 0.001, and 0.01, respectively, compared to the HCHF+MCAO+vehicle group). In addition, MCAO also decreased retention time in metabolic rats which received vehicle at 14 and 21 days after MCAO (*P* value < 0.001 and 0.05, respectively, compared to the HCHF+sham operation+vehicle group). These changes were also mitigated both at 14 and 21 days after MCAO (*P* value < 0.001 all, compared to the HCHF+MCAO+vehicle group) as shown in Figure 4. To assure that the cognitive enhancing effect observed in this study was not the false positive, the effect of OA extract on locomotor activity was also evaluated and results were shown in Figures 5 and 6. Our data showed that obese rats which subjected to MCAO and received vehicle (HCHF+MCAO+vehicle) significantly decreased the number of center square entry at days 7 and 14 after MCAO (*P* value < 0.05 all, compared to the naïve control). However, low and medium doses of OA extract could counteract the reduction of this parameter at 7 and 14 days after MCAO, respectively (*P* value < 0.01 and 0.001, respectively, compared to MCAO rats which received HCHF and vehicle). No other significant changes were observed.

### 3.3. Histological Changes in the Hippocampus

**Figure 7:**
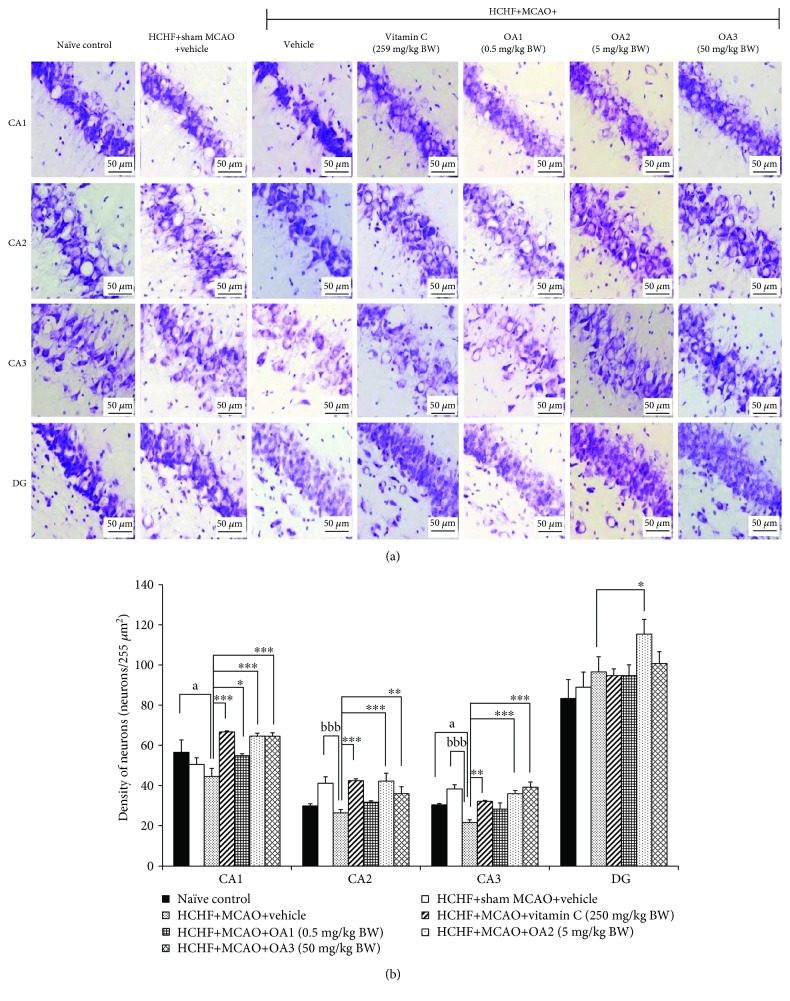
Effect of OA extract on neuron density in various subregions of the hippocampus. (a) Light microscopy of coronal sections in CA1, CA2, CA3, and dentate gyrus of the hippocampus which were stained with cresyl violet at 40x magnification. (b) Density of survival neurons in CA1, CA2, CA3, and dentate gyrus of the hippocampus. Data are presented as mean ± SEM (*n* = 6/group). ^a^*P* value < 0.05, compared to naïve intact rats; ^bbb^*P* value < 0.001, compared to sham operation which received HCHF diet and vehicle; and ^∗,∗∗,∗∗∗^*P* value < 0.05, 0.01, and 0.001, respectively, compared to MCAO rats which received HCHF and vehicle. HCHF: high-carbohydrate high-fat diet; MCAO: right middle cerebral artery occlusion; OA1, OA2, and OA3: the combined extract of *O. sativa* and *A. graveolens* at doses of 0.5, 5, and 50 mg/kg BW, respectively.

Since our data demonstrated that OA extract significantly improved spatial memory, the effect of OA extract in the hippocampus, the area contributing an important role on spatial memory [26], was also explored.  Figure 7 showed that MCAO significantly decreased neuron density in CA2 and CA3 (*P* value < 0.001 all, compared to the HCHF+sham operation+vehicle group). Vitamin C significantly increased neuron densities in CA1, CA2, and CA3 (*P* value < 0.001, 0.001, and 0.01, respectively, compared to the HCHF+MCAO+vehicle group). It was found that the medium dose of OA extract produced the significant increase in neuron densities in CA1, CA2, CA3, and dentate gyrus (*P* value < 0.001, 0.001, 0.001, and 0.05, respectively, compared to the HCHF+MCAO+vehicle group). The low dose of extract significantly increased neuron density in CA1 while the high dose of extract showed the significant increase in neuron density in both CA2 and CA3 (*P* value < 0.01 and 0.001, respectively, compared to the HCHF+MCAO+vehicle group).

**Figure 8:**
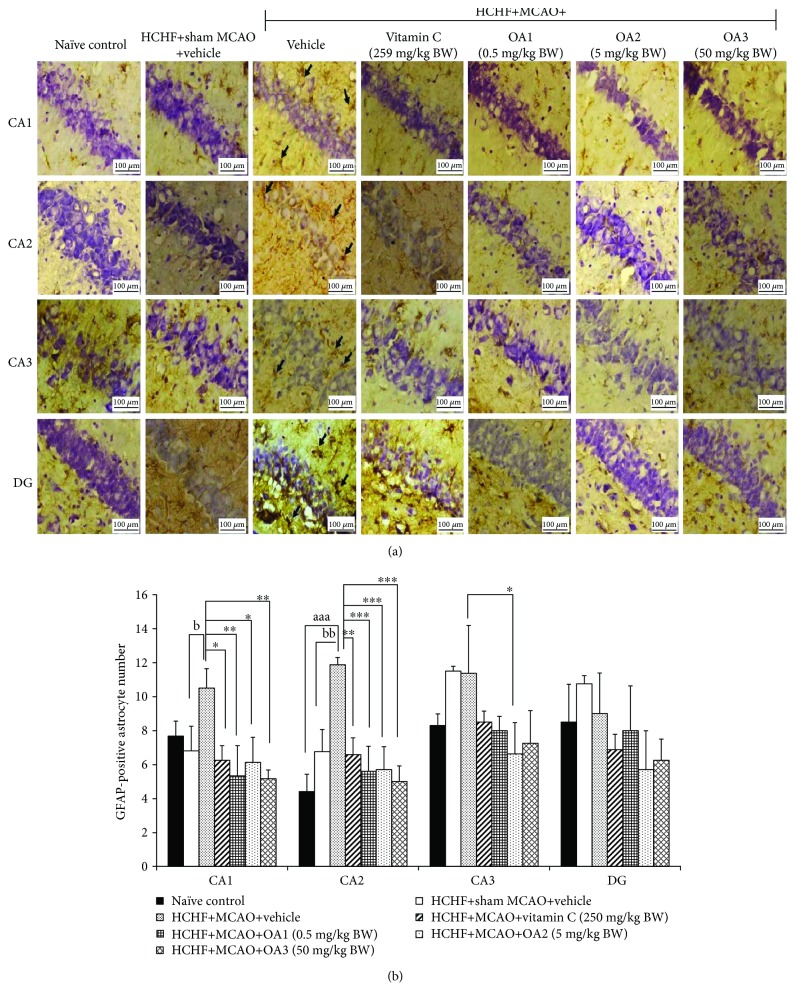
Effect of various doses of OA extract on the density of GFAP-positive cell. (a) Immunostaining for GFAP-positive cell in the hippocampus. GFAP-positive cell or astrocytes were stained brown (arrow). Magnification, 40x; scale bar = 100 *μ*m. (b) GFAP-positive cells in CA1, CA2, CA3, and dentate gyrus of the hippocampus. Data are presented as mean ± SEM (*n* = 6/group). ^aaa^*P* value < 0.001, compared to naïve intact rats; ^b,bb^*P* values < 0.05 and 0.01, respectively, compared to HCHF+MCAO+vehicle; and ^∗,∗∗,∗∗∗^*P* values < 0.05, 0.01, and 0.001, respectively, compared to MCAO rats which received HCHF and vehicle. HCHF: high-carbohydrate high-fat diet; MCAO: right middle cerebral artery occlusion; OA1, OA2, and OA3: the combined extract of *O. sativa* and *A. graveolens* at doses of 0.5, 5, and 50 mg/kg BW, respectively; GFAP: glial fibrillary acidic protein.

Figure 8 showed that a HCHF diet failed to produce the significant changes in GFAP-positive cell in the hippocampus. MCAO significantly enhanced GFAP-positive cell in CA1 and CA2 metabolic syndrome rats (*P* value < 0.05 and 0.01, respectively, compared to the HCHF+sham operation+vehicle group). Vitamin C treatment significantly mitigated the changes mentioned earlier (*P* value < 0.05 and 0.01, respectively, compared to the HCHF+MCAO+vehicle group). Interestingly, the low and high doses of OA produced the significant reduction of GFAP-positive cell densities in CA1 (*P* value < 0.01 all, compared to the HCHF+MCAO+vehicle group) and CA2 (*P* value < 0.001 all, compared to the HCHF+MCAO+vehicle group). The medium dose of OA could suppress GFAP-positive cell densities in CA1, CA2, and CA3 (*P* value < 0.05, 0.001, and 0.05, respectively, compared to the HCHF+MCAO+vehicle group).

### 3.4. Effect of OA Extract on Biochemical Parameters

**Figure 9:**
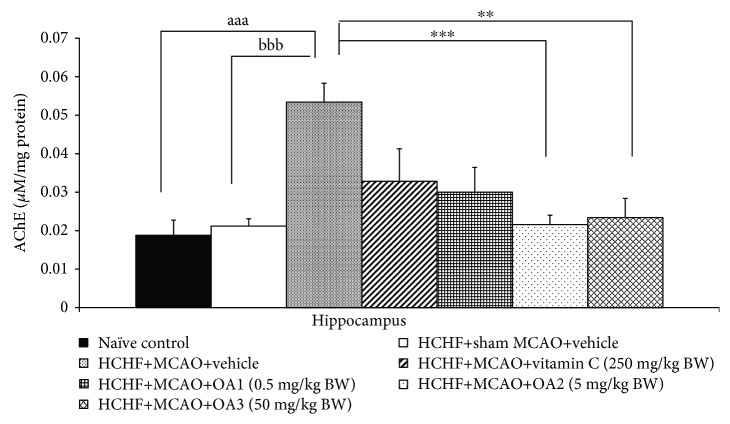
The effect of various doses of OA extract on acetylcholinesterase activity in the hippocampus. Data are presented as mean ± SEM (*n* = 6/group). ^aaa^*P* value < 0.001, compared to naïve intact rats; ^bbb^*P* value < 0.001, compared to sham operation which received HCHF diet and vehicle; and ^∗∗,∗∗∗^*P* value < 0.01 and 0.001, respectively, compared to MCAO rats which received HCHF and vehicle. HCHF: high-carbohydrate high-fat diet; MCAO: right middle cerebral artery occlusion; OA1, OA2, and OA3: the combined extract of *O. sativa* and *A. graveolens* at doses of 0.5, 5, and 50 mg/kg BW, respectively.

Based on the crucial role of acetylcholinesterase suppression on the memory-enhancing effect [17, 39–43], we also determined the effect of OA extract on AChE activity in the hippocampus and data were shown in Figure 9. MCAO significantly enhanced AChE activity in the hippocampus of metabolic syndrome rats induced by a HCHF diet which received vehicle (*P* value < 0.001, compared to the HCHF+sham operation+vehicle group). However, this change was mitigated by low and medium doses of OA extract (*P* value < 0.001 and 0.01, respectively, compared to the HCHF+MCAO+vehicle group). Vitamin C and high dose of OA extract failed to modulate AChE activity in the hippocampus.

The effect of OA extract on oxidative stress markers including malondialdehyde (MDA), superoxide dismutase (SOD), catalase (CAT), and glutathione peroxidase (GSH-Px) was also determined, and data were shown in Table 2. Sham operation and vehicle failed to produce the significant changes of the aforementioned parameters. MCAO significantly decreased SOD and GSH-Px activities but increased the MDA level (*P* value < 0.01, 0.001, and 0.001, respectively, compared to the HCHF+sham operation+vehicle group). Vitamin C mitigated the reduction of GSH-Px and the increase in the MDA level in metabolic syndrome rats subjected to MCAO (*P* value < 0.001 all, compared to the HCHF+MCAO+vehicle group). All doses of OA extract produced the significant reduction in the MDA level in metabolic syndrome rats with cerebral ischemia (*P* value < 0.001 all, compared to the HCHF+MCAO+vehicle group). However, the significant increase in SOD and GSH-Px activities was observed only in metabolic syndrome rats with cerebral ischemia which received OA extract at dose of 50 mg/kg BW (*P* value < 0.05 and 0.01, respectively, compared to the HCHF+MCAO+vehicle group).

**Figure 10:**
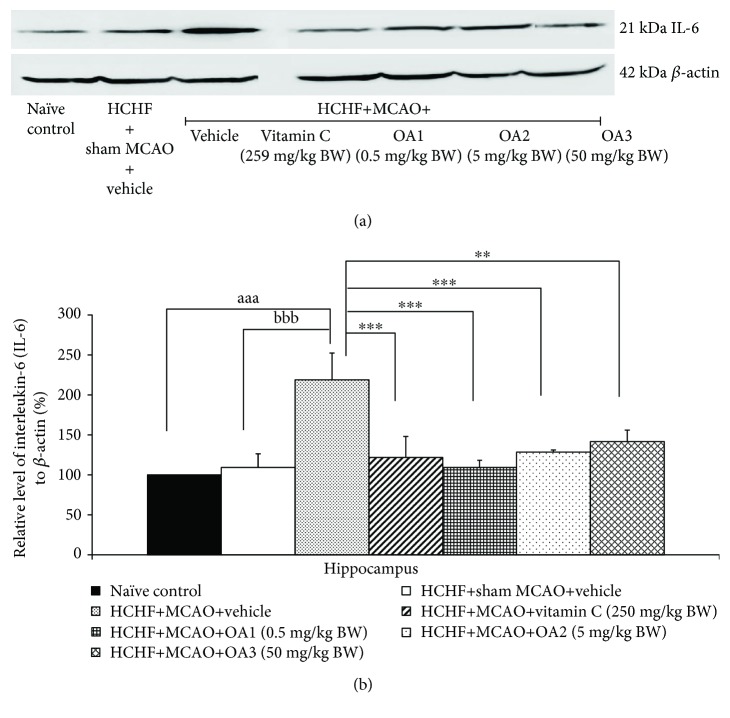
Effect of various doses of OA extract on the expression of IL-6 in the hippocampus. (a) Representative western blot showing the levels of IL-6. (b) Relative density of IL-6. Data are presented as mean ± SEM (*n* = 6/group). ^aaa^*P* value <0.001, compared to naïve intact rats; ^bbb^*P* value < 0.001, compared to sham operation which received HCHF diet and vehicle; and ^∗∗,∗∗∗^*P* value < 0.01 and 0.001, respectively, compared to MCAO rats which received HCHF and vehicle. HCHF: high-carbohydrate high-fat diet; MCAO: right middle cerebral artery occlusion; OA1, OA2, and OA3: the combined extract of *O. sativa* and *A. graveolens* at doses of 0.5, 5, and 50 mg/kg BW, respectively.

**Figure 11:**
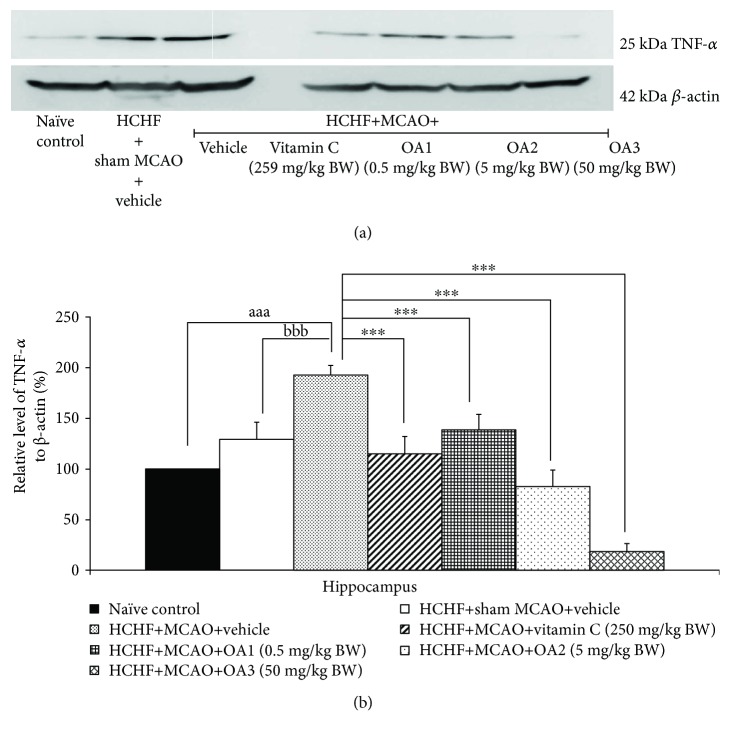
Effect of various doses of OA extract on the expressions of TNF-*α* in the hippocampus. (a) Representative western blot showing the levels of TNF-*α*. (b) Relative density of TNF-*α*. Data are presented as mean ± SEM (*n* = 6/group). ^aaa^*P* value <0.001, compared to naïve intact rats; ^bbb^*P* value <0.001, compared to sham operation which received HCHF diet and vehicle; and ^∗∗∗^*P* value < 0.001, compared to MCAO rats which received HCHF and vehicle. HCHF: high-carbohydrate high-fat diet; MCAO: right middle cerebral artery occlusion; OA1, OA2, and OA3: the combined extract of *O. sativa* and *A. graveolens* at doses of 0.5, 5, and 50 mg/kg BW, respectively.

In addition, sham operation failed to produce the significant change of interleukin-6 (IL-6) and tumor necrosis factor-*α* (TNF-*α*) levels in metabolic syndrome rats with cerebral ischemia but MCAO significantly increased the levels of both substances mentioned earlier in the hippocampus (*P* value < 0.001 all, compared to the HCHF+sham operation+vehicle group). These changes were mitigated by vitamin C and all doses of OA extract as shown in Figures 10 and 11.

## 4. Discussion

The current data clearly demonstrated that MetS rats induced by a HCHF diet failed to produce a significant increase in the MDA level and no significant reduction in antioxidant enzyme activities was observed in the hippocampus. In addition, no significant changes in TNF-*α*, IL-6, and hippocampal damage in various regions were observed. Reperfusion after cerebral ischemia (R/I) in MetS rats increased MDA, TNF-*α*, and IL-6 levels, but it decreased the activities of the main scavenger enzymes including SOD, CAT, and GSH-Px and neuron densities in CA2 and CA3 subregions of the hippocampus. These findings were in agreement with the previous studies which showed that R/I enhanced oxidative stress [44] which in turn destroyed the hippocampus, an area playing an important role in learning and memory [45, 46] leading to memory impairment [47]. It has been found that vitamin C and all doses of OA significantly decreased the MDA level, an oxidative stress damage biomarker. However, no tight association between the concentrations of OA used in this study, and the alterations of antioxidant enzyme activities were observed. Although the reduction of the MDA level was observed at all doses used in this study, the elevation of antioxidant enzyme activity was observed only at high dose of OA. These data suggested that other factors might play a role on the reduction of oxidative stress. The recent study has demonstrated that the anthocyanins can act as antioxidant and remove the oxidative stress reactive species produced during reperfusion injury directly [48]. It also enhances glutathione but decreases the MDA level together with the increase in spatial memory [49]. In addition, it can also improve mitochondrial function leading to the reduction of oxidative stress production [50]. Based on the aforementioned effect of OA on oxidative stress changes and the positive modulation effect of anthocyanins mentioned earlier, we do suggest that the reduction of oxidative stress observed in this study may be explained partly via the direct antioxidant effect of anthocyanins in OA and the elevation of GSH-Px activity which in turn induces the reduction of the MDA level induced by anthocyanins. In addition, the effect to improve mitochondria functions induced by anthocyanins may also contribute a role on the reduction of oxidative stress observed in this study.

In addition to the oxidative stress, neuroinflammation also plays the role on the brain damage and memory deficit following ischemic injury [51]. Our data also showed that reperfusion injury following cerebral ischemia in metabolic syndrome rats increased TNF-*α* and IL-6 together with the cognitive impairment which were in agreement with the previous study [51–53]. After injury, injured neurons in the core and penumbra of the lesion and glial cells in the core produce proinflammatory mediators, cytokines, and reactive oxygen species, which activate both astrocytes and microglia [54]. Then, activated astrocytes can produce the proinflammatory cytokines including IL-6, TNF*α*, and others [55, 56]. The elevations of cytokines produce the detrimental effect to brain by inducing apoptosis and suppressing the hippocampal neurogenesis [57, 58]. However, the brain damage and the neuroinflammation can be attenuated by anthocyanins [59, 60]. In addition, anthocyanins can also decrease neuroinflammation and the density of GFAP-positive cell in the hippocampus and improve memory dysfunction [61]. In addition, our data also showed that OA suppressed AChE in the hippocampus of metabolic syndrome rats.

Based on all data mentioned earlier, we suggest that anthocyanins present in OA may exert the beneficial effect at multitarget sites. Anthocyanins may improve oxidative stress in the hippocampus via the increase in GSH-Px activity and the direct antioxidant effect of anthocyanins. The reduction in the oxidative stress production by mitochondria may also contribute the role, but this part still required a further investigation. In addition, anthocyanins in OA can also decrease glial cell expression in the hippocampus resulting in the decrease in neuroinflammation leading to the increase in neurogenesis in various subregions of the hippocampus. Therefore, the density of functional neurons in the hippocampus increases giving rise to the improvement of memory performance. Since both glial cell expression and neurogenesis are under the influence of the growth factor, it is possible that anthocyanins can exert the effect to modulate the expression of the growth factor which in turn decreases GFAP-positive cell or glial cell especially astrocyte but enhances neurogenesis in the hippocampus. However, this requires further exploration. Moreover, OA also suppresses AChE activity in the hippocampus which in turn enhances an available ACh giving rise to the memory enhancement.

## 5. Conclusion

**Figure 12:**
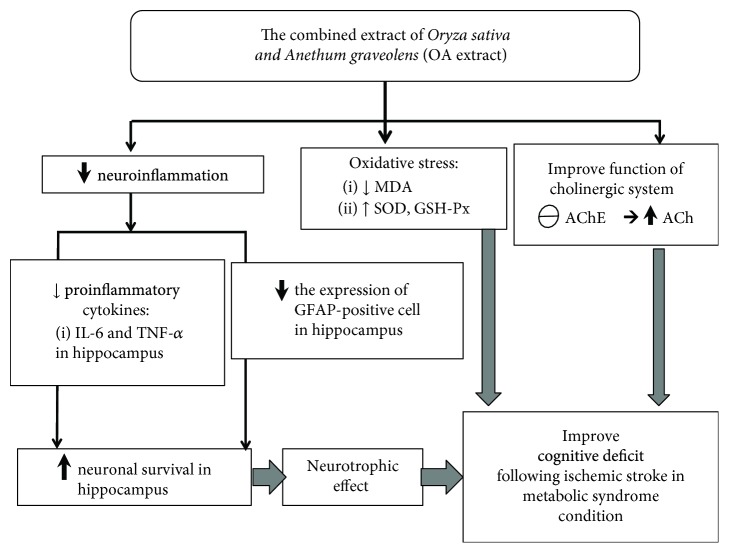
The possible underlying mechanism for the neuroprotective and cognitive enhancing effects of the combined extract of *Oryza sativa*, *L. indica*, and *Anethum graveolens* Linn. (OA extract) in an animal model of metabolic syndrome with cerebral ischemic stroke.

The current study clearly demonstrates that the combined extract of *O. sativa* and *A. graveolens* or OA is the functional ingredient to improve cognitive deficit following ischemic stroke in metabolic syndrome condition. The possible underlying mechanisms involve multitargets including the reduction of oxidative stress and neuroinflammation levels together with the suppression of AChE activity. Interestingly, OA also possesses neurotrophic effect and suppresses the expression of GFAP-positive cell in the hippocampus as shown in Figure 12. Based on the modulation effects of OA on various proteins mentioned earlier, the epigenetic modulation effect induced by OA is worth for further exploration.

## Figures and Tables

**Table 1:** Characterization of OA extract including phenolic compositions and biological activities.

Parameters	Units	*O. sativa*	*A. graveolens*	OA extract	Standard reference
Total phenolic	*μ*g GAE/mg extract	824.62 ± 90.98	941.54 ± 41.63	1724.10 ± 159.73^aaa,bb^	—
Total flavonoids	*μ*g quercetin/mg extract	47.40 ± 2.98	23.81 ± 0.32	62.18 ± 1.03^aaa,bbb^	—
*Antioxidant activities*					
DPPH	EC50 (mg/ml)	0.123 ± 0.01	0.065 ± 0.02	0.014 ± 0.003^aa^	0.008 ± 0.002, ascorbic acid
FRAP	EC50 (mg/ml)	2.56 ± 0.37	2.99 ± 0.055	1.19 ± 0.24^a,b^	2.56 ± 0.14, ascorbic acid
*Neuronal marker*					
AChE inhibition	EC50 (mg/ml)	2.03 ± 1.08	2.94 ± 0.62	1.98 ± 0.15	0.67 ± 0.001, donepezil
*Inflammatory marker*					
COX-2 inhibition	EC50 (mg/ml)	9.37 ± 1.18	142.44 ± 15.82	1.73 ± 0.44^bbb^	0.02 ± 0.001, indomethacin

Data are presented as mean ± SEM. ^a,aa,aaa^*P* value < 0.05, 0.01, and 0.001, respectively, compared with *O. sativa* and ^b,bb,bbb^*P* value < 0.05, 0.01, and 0.001, respectively, compared with *A. graveolens*. OA extract: the combined extract of *O. sativa* and *A. graveolens*.

**Table 2:** The effect of various doses of OA extract on oxidative stress markers in the hippocampus.

Treatment group	MDA level (ng/mg protein)	SOD activity (units/mg protein)	CAT activity (units/mg protein)	GSH-Px activity (units/mg protein)
Naïve control	0.16 ± 0.02	8.86 ± 1.52	4.81 ± 0.69	8.54 ± 0.59
HCHF+sham MCAO+vehicle	0.15 ± 0.04	8.73 ± 1.79	2.52 ± 0.88	10.37 ± 2.41
HCHF+MCAO+vehicle	0.89 ± 0.10^aaa,bbb^	4.73 ± 0.48^aa,bb^	0.62 ± 0.03^aaa^	3.77 ± 0.26^aa,bbb^
HCHF+MCAO+vitamin C (250 mg/kg BW)	0.37±0.08^∗∗∗^	7.47 ± 0.59	1.94 ± 0.50	6.25±0.66^∗∗∗^
HCHF+MCAO+OA1 (0.5 mg/kg BW)	0.37±0.06^∗∗∗^	6.83 ± 0.19	1.45 ± 0.21	5.60 ± 0.87
HCHF+MCAO+OA2 (5 mg/kg BW)	0.15±0.02^∗∗∗^	5.01 ± 0.40	1.67 ± 0.25	8.87 ± 0.46
HCHF+MCAO+OA3 (50 mg/kg BW)	0.25±0.07^∗∗∗^	8.01 ± 1.48^∗^	2.27 ± 0.27	8.54±0.59^∗∗^

Data are presented as mean ± SEM (*n* = 6/group). ^aa,aaa^*P* value < 0.01 and 0.001, respectively, compared to naïve intact rats; ^bb,bbb^*P* value < 0.01 and 0.001, respectively, compared to sham operation which received HCHF diet and vehicle; and ^∗,∗∗,∗∗∗^*P* value < 0.05, 0.01, and 0.001, respectively, compared to MCAO rats which received HCHF and vehicle.

## Data Availability

I confirm that data are available and will be provided on request because during this period, all data are in the process of petty patent registration.
